# 

**Published:** 1902-02-15

**Authors:** 


### CONTENTS.

The Moral Side of Dentistry (Third Paper),
*By Geo. W. Clapp, D.D.S.* 55

Nitrous Oxide and Oxygen in Dental Surgery,
*By D. H. Ziegler, D.D.S.* 66

The Cohesion of Gold, - - *By C. A. Hawley, D.D.S.* 79

Making an Inlay Matrix, - - - *By H. J. Bosart* 95

Porcelain Fillings, - - *By W. C. Capon, D.D.S.* 96

EDITORIAL, - - - - - - - - - 101

OBITUARY, - - - - - - - - - 104

ANNOUNCEMENTS, - - - - - - - - 104

INDENTS, - - - - - - - - - - 106

